# Affected neural networks as basis of disturbed motor function in schizophrenia

**DOI:** 10.1007/s00406-020-01116-z

**Published:** 2020-03-06

**Authors:** Andrea Schmitt, Daniela Reich-Erkelenz, Peter Falkai

**Affiliations:** 1 Department of Psychiatry and Psychotherapy, University Hospital, LMU Munich, Munich, Germany; 2 Institute of Psychiatric Phenomics and Genomics (IPPG), University Hospital, LMU Munich, Munich, Germany

Based on the systematic assessment of symptoms, Emil Kraepelin over 100 years ago defined catatonia and motor symptoms as a feature of schizophrenia [1]. In fact, about two-third of first-episode schizophrenia patients exhibit motor deficits; in multi-episode patients, the frequency is even higher. Irrespective of motor side-effects of antipsychotic treatment, motor slowing has been detected during the early course of psychosis. In this issue, in antipsychotic-naive individuals at high risk for psychosis (CHR), Damme et al. [2] used a computerized finger tapping task and clinical interviews. They could demonstrate motor slowing in the CHR individuals compared to healthy controls and a relationship to negative, but not positive symptoms. Accordingly, in medicated young schizophrenia patients without extrapyramidal symptoms, better fine motor function was correlated with less-severe negative symptoms [3].

Neurological soft signs (NSS) refer to minor deficits in motor coordination, sensory integration, and disinhibition, and are highly prevalent in patients with schizophrenia, but also in those with bipolar or obsessive–compulsive disorder. In a large sample of patients with psychosis, motor dysfunction at baseline has also been related to symptoms of obsessive–compulsive after a follow-up of 3 years [4], suggesting that motor symptoms may precede co-occurring obsessive–compulsive symptoms. Individuals with ultra-high risk for psychosis have been shown to exhibit a higher prevalence of NSS sensory integration items than individuals with schizotypy and healthy controls, and these items could discriminate individuals at high risk from healthy controls with an accuracy of about 85% [5]. Therefore, NSS have been proposed as biomarkers to detect and to discriminate individuals in different stages of psychosis from healthy controls. In a large sample of patients with schizophrenia, their unaffected first-degree relatives, individuals with schizotypal personality disorder, other psychiatric patients and healthy controls, and patients along the schizophrenia continuum showed increased levels of NSS compared to healthy controls and other psychiatric patients. The abnormal developmental trajectory of NSS in schizophrenia supports the endophenotype hypothesis and relates NSS to neurodevelopmental disturbances [6].

In remitted schizophrenia patients, Feng et al. [7] investigated NSS and their association with cognitive deficits. They found that six subitems of NSS and neurocognitive deficits met criteria of endophenotype and hypothesized that covariation of sensory integration and cognitive domains including information processing speed, attention, and social cognition suggest an overlap of compromised underlying neural systems. NSS have been reported to be associated with disturbed cortical–subcortical–cerebellar circuitry in schizophrenia. Using a graph theoretical approach and regional network analysis, it has been demonstrated that NSS were associated with betweenness centrality involving the inferior orbital frontal cortex, the middle temporal cortex, the hippocampus, the supramarginal cortex, the amygdala, and the cerebellum. Global network analysis revealed NSS to be associated with the distribution of network hubs involving the superior medial frontal cortex, the temporal cortices, the postcentral cortex, the amygdala, and the cerebellum [8]. In individuals with ultra-high-risk for psychosis, NSS scores were associated with decreased gray matter volume in the superior and medial frontal cortex, the rectal cortex, the pre- and postcentral cortex, the insula, the caudate, and the cerebellum [9]. The cerebellum is well known to be involved in motor control, but functional magnetic resonance imaging (fMRI) and positron emission tomography (PET) studies have also shown the involvement of the cerebellum in cognition. The cerebellum has been implicated in the pathophysiology of schizophrenia with the cortico-thalamo-cerebellar circuit receiving particular attention. However, the underlying mechanisms are unknown and are hypothesized to involve a glutamatergic deficit in cerebellar subregions of schizophrenia patients [10]. In future studies, multimodal neuroimaging should be applied to assess affected brain circuits and the pathophysiological basis of NSS during lifespan in individuals with psychosis.
